# Supplementary material for: Using antibodies to control DNA-templated chemical reactions
Source: Nat Commun. 2020 Dec 7;11:6242. doi: 10.1038/s41467-020-20024-3 (PMC7721721; doi:10.1038/s41467-020-20024-3)
Supplement: Supplementary file 1 — Reporting Summary [file 41467_2020_20024_MOESM1_ESM.pdf]

nature research

Corresponding author(s): Francesco Ricci

Last updated by author(s): Oct 1, 2020

Reporting Summary

Nature Research wishes to improve the reproducibility of the work that we publish. This form provides structure for consistency and transparency in reporting. For further information on Nature Research policies, see our Editorial Policies and the Editorial Policy Checklist.

Statistics

For all statistical analyses, confirm that the following items are present in the figure legend, table legend, main text, or Methods section.

n/a	Confirmed	
☐	☒	The exact sample size (n) for each experimental group/condition, given as a discrete number and unit of measurement
☐	☒	A statement on whether measurements were taken from distinct samples or whether the same sample was measured repeatedly
☒	☐	The statistical test(s) used AND whether they are one- or two-sided<br>*Only common tests should be described solely by name; describe more complex techniques in the Methods section.*
☒	☐	A description of all covariates tested
☒	☐	A description of any assumptions or corrections, such as tests of normality and adjustment for multiple comparisons
☐	☒	A full description of the statistical parameters including central tendency (e.g. means) or other basic estimates (e.g. regression coefficient) AND variation (e.g. standard deviation) or associated estimates of uncertainty (e.g. confidence intervals)
☒	☐	For null hypothesis testing, the test statistic (e.g. F, t, r) with confidence intervals, effect sizes, degrees of freedom and P value noted<br>*Give P values as exact values whenever suitable.*
☒	☐	For Bayesian analysis, information on the choice of priors and Markov chain Monte Carlo settings
☒	☐	For hierarchical and complex designs, identification of the appropriate level for tests and full reporting of outcomes
☒	☐	Estimates of effect sizes (e.g. Cohen's d, Pearson's r), indicating how they were calculated

Our web collection on statistics for biologists contains articles on many of the points above.

Software and code

Policy information about availability of computer code

Data collection	In vitro fluorescence data were recorded using the commercial Cary Eclipse software (version 1.2).<br>Gels from electrophoresis were imaged using Image Lab software from Biorad (version 6.0.1).
Data analysis	The design of sequences was performed using NUPACK web application available at http://www.nupack.org (version published in J Comput Chem 32: 170–173, 2011).<br>Densitometric analysis of gels from electrophoresis was performed by using Image Lab software from Biorad (version 6.0.1).

For manuscripts utilizing custom algorithms or software that are central to the research but not yet described in published literature, software must be made available to editors and reviewers. We strongly encourage code deposition in a community repository (e.g. GitHub). See the Nature Research guidelines for submitting code & software for further information.

Data

Policy information about availability of data

All manuscripts must include a data availability statement. This statement should provide the following information, where applicable:

- Accession codes, unique identifiers, or web links for publicly available datasets
- A list of figures that have associated raw data
- A description of any restrictions on data availability

Source data are provided with this paper. Data are available upon request from the corresponding author.

Field-specific reporting

Please select the one below that is the best fit for your research. If you are not sure, read the appropriate sections before making your selection.

[x] Life sciences [ ] Behavioural & social sciences [ ] Ecological, evolutionary & environmental sciences

For a reference copy of the document with all sections, see nature.com/documents/nr-reporting-summary-flat.pdf

Life sciences study design

All studies must disclose on these points even when the disclosure is negative.

Sample size	No sample-size calculations were used. Enough replicate measurements or samples were analyzed to determine that the data were sufficiently reproducible.
Data exclusions	No data exclusion.
Replication	The experiments were performed three times to ensure reproducibility of results.
Randomization	This study only contains in vitro data. Therefore, sample randomization was not relevant to this study.
Blinding	This study only contains in vitro data. Therefore, blinding was not relevant to this study.

Reporting for specific materials, systems and methods

We require information from authors about some types of materials, experimental systems and methods used in many studies. Here, indicate whether each material, system or method listed is relevant to your study. If you are not sure if a list item applies to your research, read the appropriate section before selecting a response.

Materials & experimental systems

n/a	Involved in the study	
[ ]	[x]	Antibodies
[x]	[ ]	Eukaryotic cell lines
[x]	[ ]	Palaeontology and archaeology
[x]	[ ]	Animals and other organisms
[x]	[ ]	Human research participants
[x]	[ ]	Clinical data
[x]	[ ]	Dual use research of concern

Methods

n/a	Involved in the study	
[x]	[ ]	ChIP-seq
[x]	[ ]	Flow cytometry
[x]	[ ]	MRI-based neuroimaging

Antibodies

Antibodies used	Sheep polyclonal Anti-DIG antibody (Roche Diagnostic Corporation, cat#: 11333089001); Mouse monoclonal Anti-DNP antibody (Sigma-Aldrich, cat#: D8406); Murine monoclonal anti-HIV antibody (Zeptometrix Corporation, cat#: 0801077); Anti-DIG Fab Fragment (Roche Diagnostic Corporation cat#: 11214667001).
Validation	Information regarding validation and application of the antibodies used can be found in the manufacturers' website. Sheep polyclonal Anti-DIG antibody was obtained after immunization with digoxigenin, purified by ion-exchange chromatography, and the specific IgG isolated by immunosorption; Anti-DNP antibody has been affinity purified; Anti-HIV antibody has been purified from serum-free culture supernatant by protein G chromatography; Anti-DIG Fab fragments: After immunization with digoxigenin, sheep IgGs are purified by ion exchange chromatography. Specific IgGs are isolated by immunosorption. Fab fragments obtained after papain digestion are purified by gel filtration and lyophilized.
