# Supplementary material for: Using antibodies to control DNA-templated chemical reactions
Source: Nat Commun. 2020 Dec 7;11:6242. doi: 10.1038/s41467-020-20024-3 (PMC7721721; doi:10.1038/s41467-020-20024-3)
Supplement: Supplementary file 2 — Supplementary Info [file 41467_2020_20024_MOESM2_ESM.pdf]

Supplementary Information:

Using antibodies to control DNA-templated chemical reactions

Lorena Baranda Pellejero[1], Malihe Mahdifar[1], Gianfranco Ercolani[1], Jonathan Watson[2], Tom Brown Jr[2] and Francesco Ricci[1,*]

[1] Chemistry Department, University of Rome, Tor Vergata, Via della Ricerca Scientifica, 00133, Rome, Italy;

[2] ATDBio Ltd, Magdalen Centre, Oxford Science Park, Robert Robinson Avenue, OX4 4GA, United Kingdom

*Corresponding author: francesco.ricci@uniroma2.it

Supplementary Material

Supplementary Text 1

Supplementary Figures 1–25

Supplementary Tables 1–9

Supplementary References 1–3

1. Oligonucleotide sequences

HPLC purified oligonucleotides were purchased from IBA (Gottingen, Germany), LGC Biosearch Technologies (Risskov, Denmark) or Eurofins (Ebersberg, Germany). Below are reported the sequences used.

1.1. Sequences to study the effect of the Ab-mimic strand on the duplex formation (Figure 2a)

Name	Sequence (5'-3')
F-strand#1 6 nt	**Cy3**- T**GG TAC G**TT GTT TTT GTG GGT TTT TTT TTT *AGA ATA AAA CGC CAC TG*
F-strand#1 8 nt	**Cy3**- T**GG TAC GAA** GTT TTT GTG GGT TTT TTT TTT *AGA ATA AAA CGC CAC TG*
F-strand#1 10 nt	**Cy3**- T**GG TAC GAA TT**T TTT GTG GGT TTT TTT TTT *AGA ATA AAA CGC CAC TG*
F-strand#1 12 nt	**Cy3**- T**GG TAC GAA TTC G**TT GTG GGT TTT TTT TTT *AGA ATA AAA CGC CAC TG*
F-strand#1 14 nt	**Cy3**- T**GG TAC GAA TTC GAC** GTG GGT TTT TTT TTT *AGA ATA AAA CGC CAC TG*
F-strand#1 16 nt	**Cy3**- T**GG TAC GAA TTC GAC TC**G GGT TTT TTT TTT *AGA ATA AAA CGC CAC TG*
Q-strand#2	<u>GTC ACC GCA AAA TAA GA</u> TTT TTT TTT TGA GTC G**AA TTC GTA CC** -**Cy5**
Ab-mimic strand	*CAG TGG CGT TTT ATT CT* TTT TTT TTT TTT TTT <u>TCT TAT TTT GCG GTG AC</u>

In the above sequences the portion in **bold** represents the duplex forming domain for each templating pair, while the portions in *italics* and <u>underlined</u> represent the domains complementary to the Ab-mimic strand. F-strand #1 variants were all labeled with Cy3 at the 5' end while Q-strand #2 was labeled with Cy5 at the 3' end.

1.2. Sequences to study the effect of Anti-DIG antibody on the duplex formation (Supplementary Figure 9)

Name	Sequence (5'-3')
F-strand#1 6 nt	**Cy3**- T**GG TAC G**TT GTT TTT GTG GGT TTT TTT TTT *AGA ATA AAA CGC CAC TG*
F-strand#1 8 nt	**Cy3**- T**GG TAC GAA** GTT TTT GTG GGT TTT TTT TTT *AGA ATA AAA CGC CAC TG*
F-strand#1 10 nt	**Cy3**- T**GG TAC GAA TT**T TTT GTG GGT TTT TTT TTT *AGA ATA AAA CGC CAC TG*
F-strand#1 12 nt	**Cy3**- T**GG TAC GAA TTC G**TT GTG GGT TTT TTT TTT *AGA ATA AAA CGC CAC TG*
F-strand#1 14 nt	**Cy3**- T**GG TAC GAA TTC GAC** GTG GGT TTT TTT TTT *AGA ATA AAA CGC CAC TG*
F-strand#1 16 nt	**Cy3**- T**GG TAC GAA TTC GAC TC**G GGT TTT TTT TTT *AGA ATA AAA CGC CAC TG*
DIG-Q-strand#2	**DIG** – GTC ACC GCA AAA TAA GA TTT TTT TTT T**GA GTC GAA TTC GTA CC -Cy5**
DIG-strand#3	**DIG** – TTT TTC *AGT GGC GTT TTA TTC T*

In the above sequences the portion in **bold** represents the duplex forming domain for each templating pair. The portions in *italics* in the F-strand#1 variants represent the domains complementary to DIG-strand#3 (see Supplementary Figure 9). In these and the following oligonucleotides, DIG was introduced via EDC/NHS coupling to an amine attached via a 5-carbon linker on the 5' end. F-strand #1 variants were all labeled with Cy3 at the 5' end while DIG-Q-strand #2 was labeled with Cy5 at the 3' end.

1.3. Sequences for templated reactions

Sequences used for templated click reactions were modified with azide and alkyne group, whereas the ones used for phosphoramidate reaction were modified with amine and phosphate group. Below are reported the sequences for each system.

1.3.1 Ab-mimic strand-directed CuAAC reaction (Figure 2d)

**Name**	**Sequence (5'-3')**
Strand#1 6 nt	**Azide-dT-C6**- T**GG TAC G**TT GTT TTT GTG GGT TTT TTT TTT *AGA ATA AAA CGC CAC TG*
Strand#1 8 nt	**Azide-dT-C6**- T**GG TAC GAA** GTT TTT GTG GGT TTT TTT TTT *AGA ATA AAA CGC CAC TG*
Strand#1 10 nt	**Azide-dT-C6**- T**GG TAC GAA TT**T TTT GTG GGT TTT TTT TTT *AGA ATA AAA CGC CAC TG*
Strand#1 12 nt	**Azide-dT-C6**- T**GG TAC GAA TTC G**TT GTG GGT TTT TTT TTT *AGA ATA AAA CGC CAC TG*
Strand#1 14 nt	**Azide-dT-C6**- T**GG TAC GAA TTC GAC** GTG GGT TTT TTT TTT *AGA ATA AAA CGC CAC TG*
Strand#1 16 nt	**Azide-dT-C6**- T**GG TAC GAA TTC GAC TC**G GGT TTT TTT TTT *AGA ATA AAA CGC CAC TG*
Strand#2	GTC ACC GCA AAA TAA GA TTT TTT TTT TGA GTC G**AA TTC GTA CC -Alkyne**
Ab-mimic strand	*CAG TGG CGT TTT ATT CT* TTT TTT TTT TTT TTT TCT TAT TTT GCG GTG AC

In the above sequences the portion in **bold** represents the duplex forming domain for each templating pair, while the portions in *italics* and underlined represent the domains complementary to the Ab-mimic strand. Strand #1 variants were modified at the 5' end with a thymine containing an azide group (azide-dT) linked to the oligonucleotide through a six-carbon spacer. An alkyne group was introduced at the 3′ end of the strand#2 through the use of alkyne-Modifier Serinol CPG.

1.3.2. Anti-DIG antibody-directed CuAAC reaction (Figure 3a)

**Name**	**Sequence (5'-3')**
Strand#1 6 nt	**Azide-dT-C6**- T**GG TAC G**TT GTT TTT GTG GGT TTT TTT TTT *AGA ATA AAA CGC CAC TG*
Strand#1 8 nt	**Azide-dT-C6**- T**GG TAC GAA** GTT TTT GTG GGT TTT TTT TTT *AGA ATA AAA CGC CAC TG*
Strand#1 10 nt	**Azide-dT-C6**- T**GG TAC GAA TT**T TTT GTG GGT TTT TTT TTT *AGA ATA AAA CGC CAC TG*
Strand#1 12 nt	**Azide-dT-C6**- T**GG TAC GAA TTC G**TT GTG GGT TTT TTT TTT *AGA ATA AAA CGC CAC TG*
Strand#1 14 nt	**Azide-dT-C6**- T**GG TAC GAA TTC GAC** GTG GGT TTT TTT TTT *AGA ATA AAA CGC CAC TG*
Strand#1 16 nt	**Azide-dT-C6**- T**GG TAC GAA TTC GAC TC**G GGT TTT TTT TTT *AGA ATA AAA CGC CAC TG*
DIG-strand#2	**DIG** - GTC ACC GCA AAA TAA GA TTT TTT TTT T**GA GTC GAA TTC GTA CC** -**Alkyne**
DIG-strand#3	**DIG** – TTT TT*C AGT GGC GTT TTA TTC T*

In the above sequences the portion in **bold** represents the duplex forming domain for each templating pair, while the portions in *italics* represent the domains complementary to DIG-strand #3.

1.3.3. Ab-mimic strand-directed phosphoramidate ligation (Supplementary Figure 19)

**Name**	**Sequence (5'-3')**
NH$_2$-strand#1 10 nt	**Amino-C6-** T**GG TAC GAA TT**T TTT GTG GGT TTT TTT TTT *AGA ATA AAA CGC CAC TG*
Phos-strand#2	GTC ACC GCA AAA TAA GA TTT TTT TTT TGA GTC G**AA TTC GTA CC -Phosphate**
Ab-mimic strand	*CAG TGG CGT TTT ATT CT* TTT TTT TTT TTT TTT TCT TAT TTT GCG GTG AC

In the above sequences the portion in **bold** represents the duplex forming domain for each templating pair, while the portions in *italics* and underlined represent the domains complementary to the Ab-mimic strand. Strand #1 was modified at the 5' end with an amine group linked to a six-carbon spacer. A phosphate group was introduced at the 3′ end of the strand#2.

1.3.4. Anti-DIG antibody-directed phosphoramidate ligation (Figure 3i)

**Name**	**Sequence (5'-3')**
NH$_2$-strand#1 10 nt	**Amino-C6-** T**GG TAC GAA TT**T TTT GTG GGT TTT TTT TTT *AGA ATA AAA CGC CAC TG*
Phos-DIG-strand#2	**DIG-** *GTC ACC GCA AAA TAA GA*T TTT TTT TTT GAG TCG **AAT TCG TAC C -Phos**
DIG-strand#3	**DIG** – TTT TT*C AGT GGC GTT TTA TTC T*

In the above sequences the portion in **bold** represents the duplex forming domain for each templating pair, while the portions in *italics* represent the domains complementary to DIG-strand#3.

1.3.5. Anti-DNP antibody-directed CuAAC reaction (Figure 4a)

Name	Sequence (5'-3')
Strand#1 10 nt	**Azide dT-C6**- T**GG TAC GAA TT**T TTT GTG GGT TTT TTT TTT *AGA ATA AAA CGC CAC TG*
DNP-strand#2	**DNP** – GTC ACC GCA AAA TAA GA TTT TTT TTT TGA GTC G**AA TTC GTA CC -Alkyne**
DNP-strand#3	**DNP** – TTT TT*C AGT GGC GTT TTA TTC T*

In the above sequences the portion in **bold** represents the duplex forming domain for each templating pair, while the portions in *italics* represent the domains complementary to DNP-strand#3. DNP was introduced via a triethylene glycol (TEG) spacer arm on the 5' end of DNP-strand#2 and of DNP-strand#3.

1.3.6. Anti-HIV antibody-directed CuAAC reaction (Figure 4d)

Name	Sequence (5'-3')
Strand#1 10 nt	**AzidedT-C6**- T**GG TAC GAA TT**T TTT GTG GGT TTT TTT TTT *AGA ATA AAA CGC CAC TG*
HIV-strand#2 (frame-inverted)	3'- *GTC ACC GCA AAA TAA GA* -5' 5'- TTT TTT TTT TGA GTC G**AA TTC GTA CC -Alkyne** - 3'
HIV-strand#3 (p17-PNA sequence)*	N-term - <u>ELDRWEKIRLRP</u> - C-term – 5'- *CAG TGG CGT TTT ATT CT* - 3'
Control product 10 nt	*GTC ACC GCA AAA TAA GA*T TTT TTT TTT GAG TCG AAT TCG TAC CTGGTA CGA ATT TTT TGT GGG TTT TTT TTT T*AG AAT AAA ACG CCA CTG*

In the above sequences the portion in **bold** represents the duplex forming domain for each templating pair, while the portions in *italics* represent the domains complementary to HIV-

strand#3. HIV-strand#3 is a PNA-Peptide chimera sequence that contains a peptide portion (portion underlined refers to the amino acid sequence, standard one-letter code, and represents the anti-HIV p17 antigen sequence) terminally conjugated to PNA.

1.3.7. Orthogonal control of CuAAC reactions by anti-DIG and anti-DNP (Figure 4g)

For the orthogonal control of reactions, two different reactant strands #1 were employed, each of them conjugated to a different fluorophore (FAM and CALFluorRed-CFR- 610) at the opposite end of the azide group.

**Name**	**Sequence (5'-3')**
FAM-strand#1	**AzidedT-C6-** T**GG TAC GAA TT**T TTT GTG GGT TTT TTT TTT *AGA ATA AAA CGC CAC TG* - **C3-FAM**
DIG-strand#2	**DIG** - GTC ACC GCA AAA TAA GA TTT TTT TTT T**GA GTC GAA TTC GTA CC** -**Alkyne**
DIG-strand#3	**DIG** – TTT TT*C AGT GGC GTT TTA TTC T*
CFR610-strand#1	**AzidedT-C6-** T**GG TAC GAA TT**T TTT GTG GGT TTT TTT TTT AGA ATA AAA CGC CAC TG - **CFR610**
DNP-strand#2	**DNP** – GTC ACC GCA AAA TAA GA TTT TTT TTT TGA GTC G**AA TTC GTA CC** -**Alkyne**
DNP-strand#3	**DNP** – TTT TTC AGT GGC GTT TTA TTC T

In the above sequences the portion in **bold** represents the duplex forming domain for each templating pair, while the portions in *italics* and underlined represent the domains complementary to DIG-strand#3 and DNP-strand#3, respectively.

1.3.8. Aptamer formation mediated by anti-DIG antibody (Figure 5a)

The sequence of the thrombin-inhibiting aptamer used for this strategy originates from a previously reported study where Ikebukuro and co-workers[1] introduced a stem-loop sequence to an active 31-nt aptamer (Supplementary Figure 24). We re-designed this aptamer by removing the loop and splitting the aptamer at the end of the complementary domain. The length of the complementary portion was 6-nt and reactive groups were introduced to the strands at the end of the duplex. Moreover, at each of the opposite ends of the strands from the stem region, a complementary sequence (17-nt) was introduced to hybridize to a DIG-modified strand. This allows the antibody-induced co-localization and subsequent reaction between the splits.

**Name**	**Sequence (5'-3')**
Split#1	GTC ACC GCA AAA TAA GA TTC ACT GGT AGG TTG GTG TGG TT**G CGG AT** - **3'-Alkyne**
Split#2	**AzidedT-C6**- T**AT CCG C**TT GGG GCC AGT GTT *AGA ATA AAA CGC CAC TG*
DIG-strand#3	**DIG** – TTT TT*C AGT GGC GTT TTA TTC T*
DIG-strand#4	TCT TAT TTT GCG GTG ACT TTT T - **DIG**

In the above sequences the portion in **bold** represents the duplex forming domain for each templating pair, while the portions in *italics* and underlined represent the domains complementary to DIG-strand#3 and DIG-strand#4, respectively.

2. Dissociation constants evaluation and estimation

Binding curves between Strand#1 and Strand#2 in the absence and presence of the Ab-mimic strand were carried out for a series of complementary domains. In Figure 2b and

Supplementary Figures 1-5 are reported the binding curves for complementary domains of 10-nt and 16nt-6nt, respectively.

Analysis of the binding curves in the absence of templates

Let *strand*#1 be the strand carrying the fluorophore, *strand*#2 the strand carrying the quencher, and *duplex* the DNA duplex obtained by hybridization of *strand*#1 with *strand*#2. Then the dissociation constant of the *duplex*, $K_{D\_no\_template}$ is defined as

$$K_{D\_no\_template} = \frac{[strand\#1][strand\#2]}{[duplex]} \qquad \text{(Equation S1)}$$

Under a normalized fluorescent condition (0-1) in which only *strand*#1 is fluorescent, the normalized fluorescence is given by the molar fraction of *strand*#1.

$$\frac{F_{(strand\#1)} - F_{min}}{F_{max} - F_{min}} = \frac{[strand\#1]}{[strand\#1] + [duplex]} \qquad \text{(Equation S2)}$$

where $F_{(strand\#1)}$ is the observed fluorescence, and $F_{\min}$ and $F_{\max}$ are the minimum and maximum fluorescence values. Multiplying numerator and denominator of equation (S2) by [*strand*#2]/[*duplex*], and taking into account equation (S1), equation (S3) is obtained.

$$F_{(strand\#1)} = F_{min} + (F_{max} - F_{min})\ \frac{K_{D\_no\_template}}{K_{D\_no\_template} + [strand\#2]} \qquad \text{(Equation S3)}$$

From the analysis of binding curves in the absence of any template, the value of $K_{D\_no\_template}$ could be reliably evaluated only for the complementary domains of 10-nt ($K_{D\_no\_template}$ = 4.7×10^{-7} M) and 12-nt ($K_{D\_no\_template}$ = 7×10^{-9} M). For complementary domains shorter than 10-nt or longer than 12-nt, the dissociation constant is either too large or too small to be reliably evaluated from binding curves. For these cases, we estimated the $K_{D\_no\_template}$ values as follows. The Gibbs free energy values for DNA hybridization under standard

conditions can be predicted by available software[2], and are reported in Supplementary Table 1.

Although, as far as their absolute value is concerned, these Gibbs free energies have to be considered with caution, their trend is significant. Thus, it is reasonable to assume a linear free energy relationship between experimental log $K_{D\_no\_template}$ values and ΔG_{pred}. On the basis of the known experimental values of $K_{D\_no\_template}$ for nt 10 and nt 12, the following linear relationship can be calculated that allows the estimation of the unknown $K_{D\_no\_template}$ values under our experimental conditions.

$$\log K_{D\_no\_template} = 0.3526\Delta G_{pred} - 0.2400 \qquad \text{(Equation S4)}$$

A summary of the $K_{D\_no\_template}$ values is reported in Supplementary Table 2. Of note, the estimated $K_{D\_no\_template}$ values are consistent with the observed behaviours in the titration experiments.

Analysis of the binding curves in the presence of the Ab-mimic strand

Let us consider the dissociation constant of the templated duplex shown in Figure 2a to give the duplex and the free Ab-mimic strand

$$K_{Ab\_mimic} = \frac{[duplex][Ab\_mimic]}{[templated\ duplex]} \qquad \text{(Equation S5)}$$

Taking into account equations (S1) and (S5), we can define the apparent dissociation constant of the templated duplex in the presence of a given excess concentration of Ab-mimic strand as follows

$$K_{D\_Ab\_mimic} = K_{D\_no\_template}\frac{K_{Ab\_mimic}}{[Ab\_mimic]} = \frac{[strand\#1][strand\#2]}{[templated\ duplex]} \qquad \text{(Equation S6)}$$

Equation (S6) is reported as equation (2) in the main text.

Under a normalized fluorescent condition (0-1) in which only *strand*#1 is fluorescent, the normalized fluorescence in the presence of an excess concentration of Ab-mimic strand is given by the molar fraction of *strand*#1.

$$\frac{F_{(strand\#1)} - F_{min}}{F_{max} - F_{min}} = \frac{[strand\#1]}{[strand\#1] + [duplex] + [templated\ duplex]} \quad \text{(Equation S7)}$$

Provided that K_{Ab_mimic} /[*Ab_mimic*] < 0.05, from equation (S5) [*templated duplex*] > 20 [*duplex*]. Accordingly, equation (S7) reduces to equation (S8).

$$\frac{F_{(strand\#1)} - F_{min}}{F_{max} - F_{min}} = \frac{[strand\#1]}{[strand\#1] + [templated\ duplex]} \quad \text{(Equation S8)}$$

Multiplying numerator and denominator of equation (S8) by [*strand*#2]/[*templated duplex*], and taking into account equation (S6), equation (S9) is obtained.

$$F_{(strand\#1)} = F_{min} + (F_{max} - F_{min}) \frac{K_{D\_Ab\_mimic}}{K_{D\_Ab\_mimic} + [strand\#2]} \quad \text{(Equation S9)}$$

Owing to the similarity of equations (S3) and (S9), the two equations are summarized by equation (1) in the main text.

By the binding curves in the presence of an excess concentration of Ab-mimic strand, it was possible to evaluate $K_{D\_Ab\_mimic}$ only for the complementary domain of 10-nt (Figure 2b). From this experimental value and the known value of $K_{D\_no\_template}$ from Supplementary Table 2, a value of $K_{Ab\_mimic} = 2.2 \times 10^{-10}$ M could be calculated by equation (S6). Even if the value of K_{Ab_mimic} could be evaluated only for the complementary domain of 10-nt, it is reasonable to assume that this value is independent of the length of the complementary domain. Accordingly, by using the values of $K_{D\_no\_template}$ from Supplementary Table 2 and the value

of K_{Ab_mimic} = 2.2 × 10^{-10} M for all the lengths of the complementary domain, it is possible to calculate by equation (S6) the value of the apparent dissociation constant of the templated duplex at a given free concentration of Ab-mimic strand, e.g. for [*Ab_mimic*] = 100 nM, the data are reported in Supplementary Table 3.

Duplex molar fractions in the absence and presence of the Ab-mimic strand

It is interesting to calculate duplex molar fractions in the absence and presence of the Ab-mimic strand at the diluted conditions that will be used for subsequent templated reactions (i.e. equimolar concentration of templating pairs, and Ab-mimic strand, 100 nM).

In the absence of added template, equation (S10) holds.

$$K_{D\_no\_template} = c\frac{(1-x)^2}{x} \quad \text{(Equation S10)}$$

where *c* is the analytical concentration of *strand*#1 and *strand*#2, and *x* is the molar fraction of the *duplex* defined as *x* = [*duplex*]/*c*. Setting *a* = $K_{D\_no\_template}$ /*c*, equation (S10) is solved to give equation (S11).

$$x = (1 + a/2) - \sqrt{a + a^2/4} \quad \text{(Equation S11)}$$

In Supplementary Table 4 are reported the molar fraction calculated taking into account the values of $K_{D\_no\_template}$ from Supplementary Table 2 and equation (S12)

In the presence of equimolar concentration of templating pairs and Ab-mimic strand, 100 nM, equation (S12) holds.

$$K_{D\_no\_template}K_{Ab\_mimic} = c^2\frac{(1-x)^3}{x} \qquad \text{(Equation S12)}$$

Setting $b = K_{D\_no\_template}\ K_{Ab\_mimic}\ /c^2$, equation (S12) can be rewritten as equation (S13).

$$x^3 - 3x^2 + (3+b)x - 1 = 0 \qquad \text{(Equation S13)}$$

Values of b can be calculated from $K_{D\_no\_template}$ from Supplementary Table 2 and from $K_{Ab\_mimic} = 2.2 \times 10^{-10}$ M. Equation (S13) can be solved by algebraic calculators (for example, https://www.calculatorsoup.com/calculators/algebra/cubicequation.php). Values of molar fractions in the presence of Ab-mimic strand at 10^{-7} M are reported in Supplementary Table 4 and plotted in Supplementary Figure 6. Differences between the molar fractions in the presence and in absence of the Ab-mimic strand are also reported in Supplementary Table 4 and plotted in Figure 2c.

Binding curves between Strand#1 and Strand#2 in the absence and presence of the anti-DIG antibody were carried out for a series of complementary domains (Supplementary Figure 9). In Supplementary Figures 10-15 are reported the binding curves for complementary domains in the range 16nt-6nt. Binding curves in the absence of added templates have been discussed above.

Analysis of the binding curves in the presence of the anti-DIG antibody

The analysis of the binding curves in the presence of the anti-DIG antibody is based on the same equations already illustrated for the analysis of binding curves in the presence of the Ab mimic strand. In particular, equation (S6) and (S9) are simply rewritten to take into account the different nature of the template as equation (S14) and (S15), which are summarized in the main text by equation (2) and (1), respectively.

$$K_{D\_Anti\_DIG\_Ab} = K_{D\_no\_template} \frac{K_{Anti\_Dig\_Ab}}{[Anti\_Dig\_Ab]} = \frac{[strand\#1][strand\#2]}{[templated\ duplex]} \qquad \text{(Equation S14)}$$

$$F_{(strand\#1)} = F_{min} + (F_{max} - F_{min}) \frac{K_{D\_Anti\_Dig\_Ab}}{K_{D\_Anti\_Dig\_Ab} + [strand\#2]} \qquad \text{(Equation S15)}$$

By the binding curves in the presence of an excess concentration of anti-DIG antibody, it was possible to evaluate $K_{D\_Anti\_DIG\_Ab}$ only for the complementary domain of 10-nt (Supplementary Figure 13). From this experimental value and the known value of $K_{D\_no\_template}$ from Supplementary Table 2, a value of $K_{Anti\_DIG\_Ab} = 3.6 \times 10^{-9}$ M could be calculated by equation (S14). Even if the value of $K_{Anti\_DIG\_Ab}$ could be evaluated only for the complementary domain of 10-nt, it is reasonable to assume that this value is independent of the length of the complementary domain. Accordingly, by using the values of $K_{D\_no\_template}$ from Supplementary Table 2 and the value of $K_{Anti\_DIG\_Ab} = 3.6 \times 10^{-9}$ M for all the lengths of

the complementary domain, it is possible to calculate by equation (S14) the value of the apparent dissociation constant of the templated duplex at a given free concentration of anti-DIG antibody, e.g. for [Anti-DIG antibody] = 100 nM, the data are reported in Supplementary Table 5.

Duplex molar fractions in the absence and presence of the anti-DIG antibody

It is interesting to calculate duplex molar fractions in the absence and presence of the anti-DIG antibody at the diluted conditions that will be used for subsequent templated reactions (i.e. equimolar concentration of templating pairs, and anti-DIG antibody, 100 nM).

Actually, duplex molar fractions in the absence of any template have been already calculated by equation (S11) and reported in Supplementary Table 4. For the sake of direct comparison, they are also reported in Supplementary Table 6.

Duplex molar fractions in the presence of the anti-DIG antibody can be calculated by solving equation (S13) where b is now defined as $b = K_{D\_no\_template}\ K_{Anti\_DIG\_Ab}\ /c^2$. Values of b can be calculated from $K_{D\_no\_template}$ from Supplementary Table 2 and from $K_{Anti\_DIG\_Ab} = 3.6 \times 10^{-9}$ M. Solving equation (S13) by algebraic calculators (for example, https://www.calculatorsoup.com/calculators/algebra/cubicequation.php), gives the molar fractions in the presence of anti-DIG antibody at 10^{-7} M reported in Supplementary Table 6 and plotted in Supplementary Figure 16. The slightly lower molar fraction obtained in the case of 16-nt is probably an artifact due to the neglect of free duplex concentration. Differences between the molar fractions in the presence and in absence of the anti-DIG antibody are also reported in Supplementary Table 6 and plotted in Supplementary Figure 17.

3. Kinetic model

Consider two complementary strands, *strand*#1 and *strand*#2 with end functional groups capable of reacting irreversibly with each other to yield a covalently linked product. Let us assume that the equilibrium of hybridization of the two strands is much faster than the irreversible reaction between the functional groups. In the absence of templates, Scheme S1 holds, where $K_{D\_no\_template}$ is the dissociation constant of the duplex, and k is the kinetic constant for the intramolecular reaction between the co-localized end groups. The second order intermolecular reaction between the end functional groups has been neglected because the reactions were carried under high dilution condition.

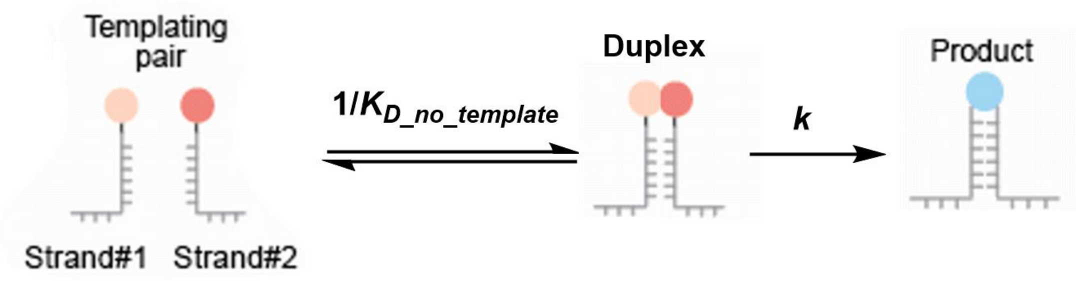

Scheme S1

Reactions were carried out at 37 °C for 2 hours with the strands at the same initial concentration of 100 nM.

The rate equation for the process depicted in Scheme S1 is given by equation (S16)

$$\frac{d[product]}{dt} = k[duplex] \qquad \text{(Equation S16)}$$

In order to integrate equation (S16), the free concentration of duplex and product must be expressed as a function of the total substrate concentration at time *t*, namely c_t. To this end, let us consider the mass balance equation

$$c_t - [duplex] = [strand\#1] \qquad \text{(Equation S17)}$$

Taking the square of equation (S17), equation (S18) is obtained

$$c_t^2 + [duplex]^2 - 2[duplex]c_t = [strand\#1]^2 \qquad \text{(Equation S18)}$$

Since [*strand*#2] = [*strand*#1], according to equation (S1), $[strand\#1]^2 = K_{D\_no\_template}\,[duplex]$. Thus, equation (S18) becomes equation (S19), that can be solved to give equation (S20).

$$[duplex]^2 - \left(K_{D\_no\_template} + 2c_t\right)[duplex] + c_t^2 = 0 \qquad \text{(Equation S19)}$$

$$[duplex] = K_{D\_no\_template}\left(0.5 + \frac{c_t}{K_{D\_no\_template}} - \sqrt{0.25 + \frac{c_t}{K_{D\_no\_template}}}\right) \qquad \text{(Equation S20)}$$

Substituting equation (S20) into equation (S16), and considering that [*product*] = $c_0 - c_t$, equation (S21) is obtained

$$-\frac{1}{K_{D\_no\_template}}\frac{dc_t}{dt} = k\left(0.5 + \frac{c_t}{K_{D\_no\_template}} - \sqrt{0.25 + \frac{c_t}{K_{D\_no\_template}}}\right) \qquad \text{(Equation S21)}$$

Setting $x = c_t / K_{D\_no\_template}$, equation (S21) becomes

$$-\frac{dx}{dt} = k\left[0.5 + x - \sqrt{0.25 + x}\right] \quad \text{(Equation S22)}$$

Separating the variables and integrating from $t = 0$ to t

$$\int_{c_0/K_{D\_no\_template}}^{c_t/K_{D\_no\_template}} -\frac{dx}{\left[0.5 + x - \sqrt{0.25 + x}\right]} = k\int_0^t dt \quad \text{(Equation S23)}$$

$$\left|\frac{1}{\left(-0.5 + \sqrt{0.25 + x}\right)} - 2\ln\left(-0.5 + \sqrt{0.25 + x}\right)\right|_{c_0/K_{D\_no\_template}}^{c_t/K_{D\_no\_template}} = kt \quad \text{(Equation S24)}$$

Setting $r_0 = -0.5 + (0.25 + c_0 / K_{D\_no\_template})^{1/2}$, and $r_t = -0.5 + (0.25 + c_t / K_{D\_no\_template})^{1/2}$, equation (S24) becomes

$$\frac{1}{r_t} - 2\ln r_t = kt + \frac{1}{r_0} - 2\ln r_0 \quad \text{(Equation S25)}$$

Dividing the equation by 2, and subtracting ln 2 to both members, equation (S26) is obtained

$$\frac{1}{2r_t} - \ln 2r_t = \frac{1}{2}kt + \frac{1}{2r_0} - \ln 2r_0 \quad \text{(Equation S26)}$$

Setting $y = 1/(2r_t)$ and taking the right-hand member equal to z, equation (S26) can be rewritten as

$$y + \ln y = z \quad \text{(Equation S27)}$$

Equation (S27) has as solution equation (S28)

$$y = \omega(z) \qquad \text{(Equation S28)}$$

where ω(z) is the Wright omega function. It can be calculated with the program MATLAB (ver. R2020a) for any value of the argument z.

Substituting the definition of y in equation (S28) and solving for c_t, equation (S29) is obtained.

$$c_t = \frac{K_{D\_no\_template}}{2\omega(z)}\left(\frac{1}{2\omega(z)} + 1\right) \qquad \text{(Equation S29)}$$

Since the percent reaction yield is defined as (c_0 – c_t)100/ c_0, from equation (S29) the reaction yield can be easily calculated as a function of c_0, $K_{D\_no\_template}$, k, and t, by equation (S30).

$$Reaction\ yield\ (\%) = \left[c_0 - \frac{K_{D\_no\_template}}{2\omega(z)}\left(\frac{1}{2\omega(z)} + 1\right)\right]\frac{100}{c_0} \qquad \text{(Equation S30)}$$

where z, the argument of the Wright omega function, is given by equation (S31).

$$z = \frac{1}{2}kt + \left[\left(1 + 4c_0/K_{D\_no\_template}\right)^{1/2} - 1\right]^{-1} - \ln\left[\left(1 + 4c_0/K_{D\_no\_template}\right)^{1/2} - 1\right]$$

(Equation S31)

When the reaction is carried out in the presence of either the Ab-mimic strand or the anti-DIG antibody, the same equation (S30) holds with the only difference that the apparent

dissociation constant $K_{D\_Ab\_mimic}$ or $K_{D\_Anti\_DIG\_Ab}$, as given by equation (S6) or (S14), substitutes $K_{D\_no\_template}$. To this end, equations (S30) and (S31) are generalized as equations (3) and (4) in the main text.

Experimental reaction yields

The 5'-azide modified (strand#1) and 3'-alkyne modified (strand#2), in the absence of any template, were mixed at a concentration of 100 nM, under the conditions specified in Methods, and left to react for 2 h. In Supplementary Table 7 are reported the experimental reaction yields together with the corresponding $K_{D\_no\_template}$ values from Supplementary Table 2.

The 5'-azide modified (strand#1), 3'-alkyne modified (strand#2) and unmodified (Ab-mimic strand) oligos were combined at a concentration of 100 nM, under the conditions specified in Methods, and left to react for 2 h. In Supplementary Table 8 are reported the experimental reaction yields, together with the calculated average free concentrations of Ab-mimic strand used to calculate $K_{D\_Ab\_mimic}$ values, by equation (S6), also reported in Supplementary Table 8.

The 5'-azide modified (strand#1), 3'-alkyne/5'-antigen modified (antigen-strand#2), and 5'-antigen modified (antigen-strand#3) oligos were combined at a concentration of 100 nM under the conditions specified in Methods. The anti-DIG antibody was added to the solution at the concentration of 300 nM and left to react for 2 h. In Supplementary Table 9 are reported the experimental reaction yields, together with the calculated average free concentrations of the anti-DIG antibody used to calculate $K_{D\_Anti\_DIG\_Ab}$ values, by equation (S14), also reported in Supplementary Table 9.

All the experimental reaction yields reported in Supplementary Tables 7-9, in the absence and in the presence of Ab-mimic strand or anti-DIG antibody, have been fitted to equation (3) in the main text so as to optimize the value of the kinetic constant k. The optimized value

of k = 0.465 h^{-1} corresponds to a maximum plateau yield of 60.5 %. In Supplementary Figure 25 are reported all the experimental points and the fitting curve calculated by equation (3) with k = 0.465 h^{-1}. In Figures 2g and 3e are reported the experimental points relative to Supplementary Tables 7 and 8, and 7 and 9, respectively, together with the same fitting curve calculated by equation (3) with k = 0.465 h^{-1}.

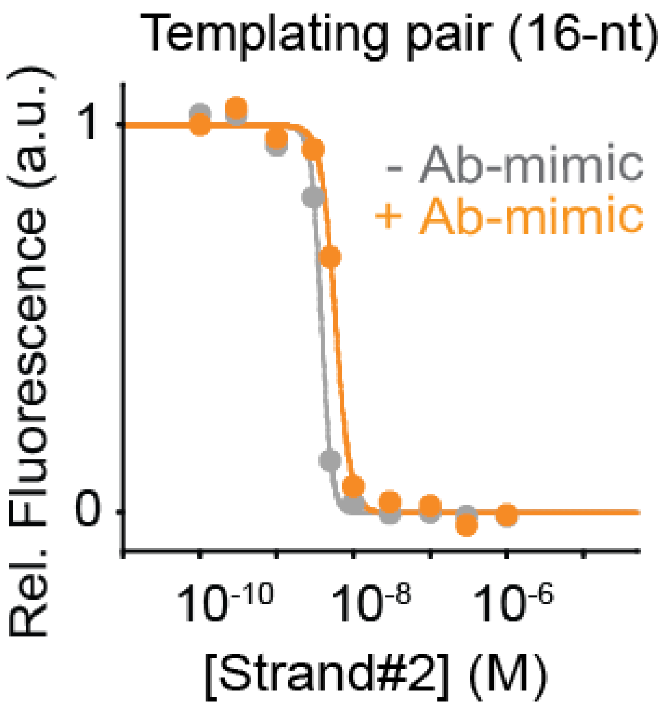

Supplementary Figure 1. ***Binding curves of the templating strands with a 16-nt complementary portion in the presence and absence of Ab-mimic strand.*** *Fluorescent experiments were performed at 37ºC in 25 mM HEPES buffer pH 7.2, 0.1 M NaCl at a fixed concentration of Strand#1 (10 nM) and increasing concentrations of Strand#2 in the absence or presence (10 nM) of Ab-mimic strand.*

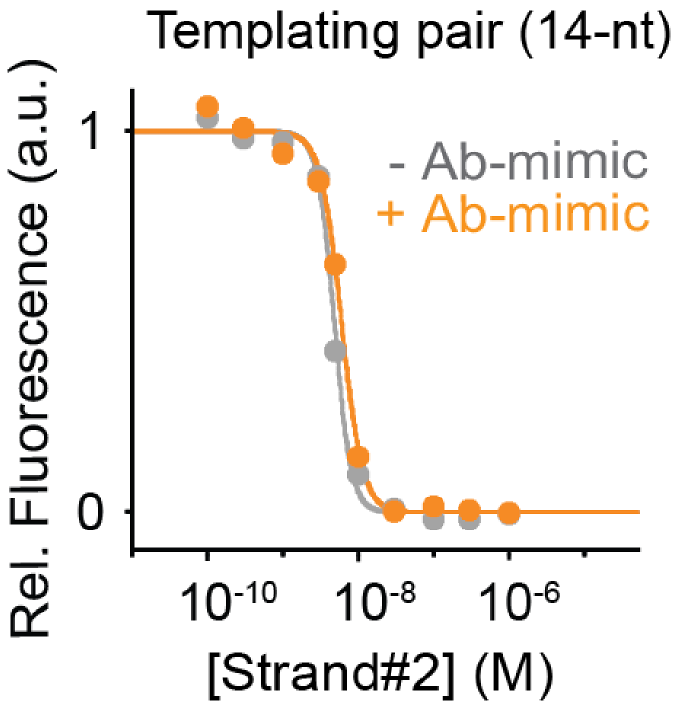

Supplementary Figure 2. ***Binding curves of the templating strands with a 14-nt complementary portion in the presence and absence of Ab-mimic strand.*** *Fluorescent experiments were performed at 37ºC in 25 mM HEPES buffer pH 7.2, 0.1 M NaCl at a fixed concentration of Strand#1 (10 nM) and increasing concentrations of Strand#2 in the absence or presence (10 nM) of Ab-mimic strand.*

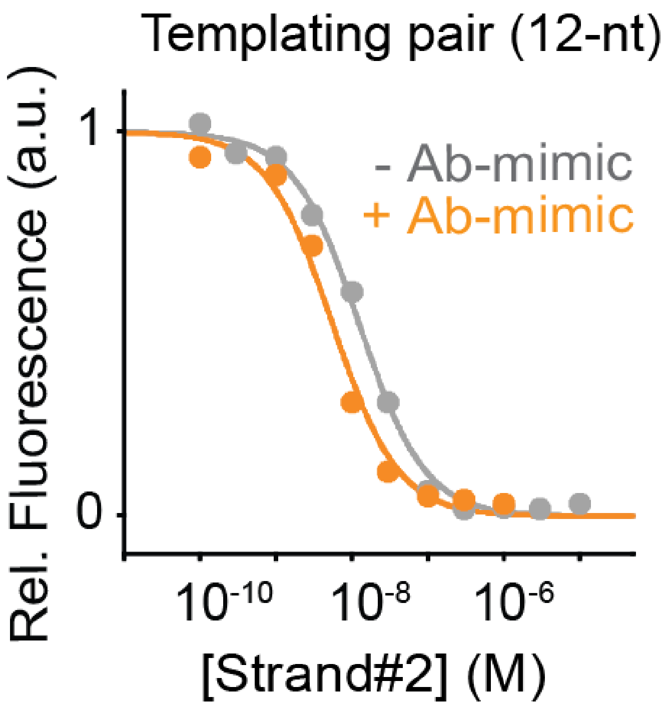

Supplementary Figure 3. ***Binding curves of the templating strands with a 12-nt complementary portion in the presence and absence of Ab-mimic strand.*** *Fluorescent experiments were performed at 37ºC in 25 mM HEPES buffer pH 7.2, 0.1 M NaCl at a fixed concentration of Strand#1 (10 nM) and increasing concentrations of Strand#2 in the absence or presence (10 nM) of Ab-mimic strand.*

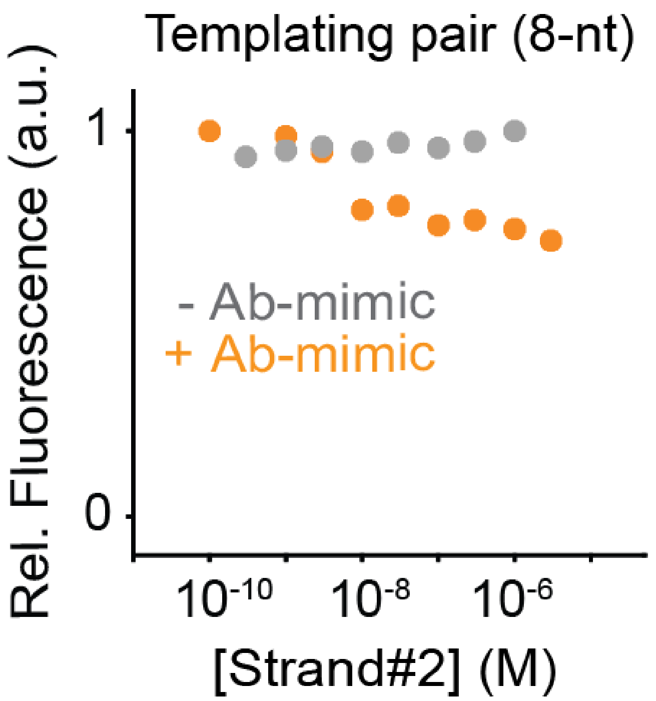

Supplementary Figure 4. *Binding curves of the templating strands with an 8-nt complementary portion in the presence and absence of Ab-mimic strand.* *Fluorescent experiments were performed at 37ºC in 25 mM HEPES buffer pH 7.2, 0.1 M NaCl at a fixed concentration of Strand#1 (10 nM) and increasing concentrations of Strand#2 in the absence or presence (10 nM) of Ab-mimic strand.*

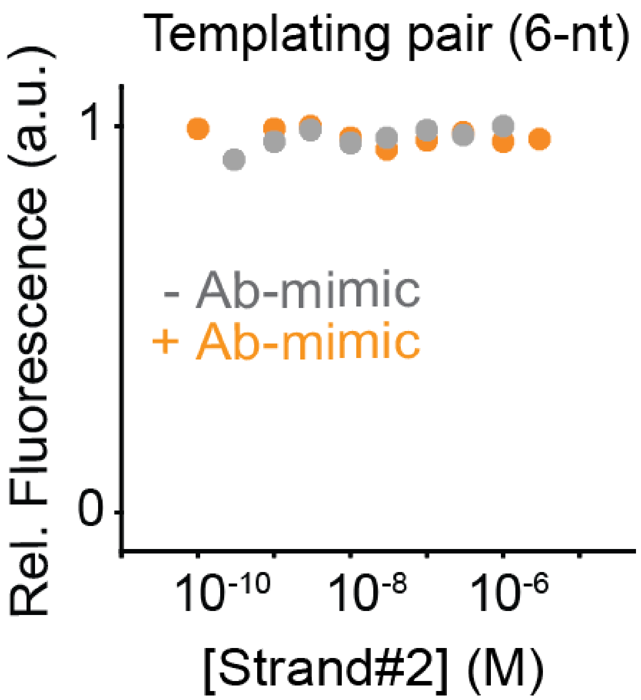

Supplementary Figure 5. ***Binding curves of the templating strands with a 6-nt complementary portion in the presence and absence of Ab-mimic strand.*** *Fluorescent experiments were performed at 37ºC in 25 mM HEPES buffer pH 7.2, 0.1 M NaCl at a fixed concentration of Strand#1 (10 nM) and increasing concentrations of Strand#2 in the absence or presence (10 nM) of Ab-mimic strand.*

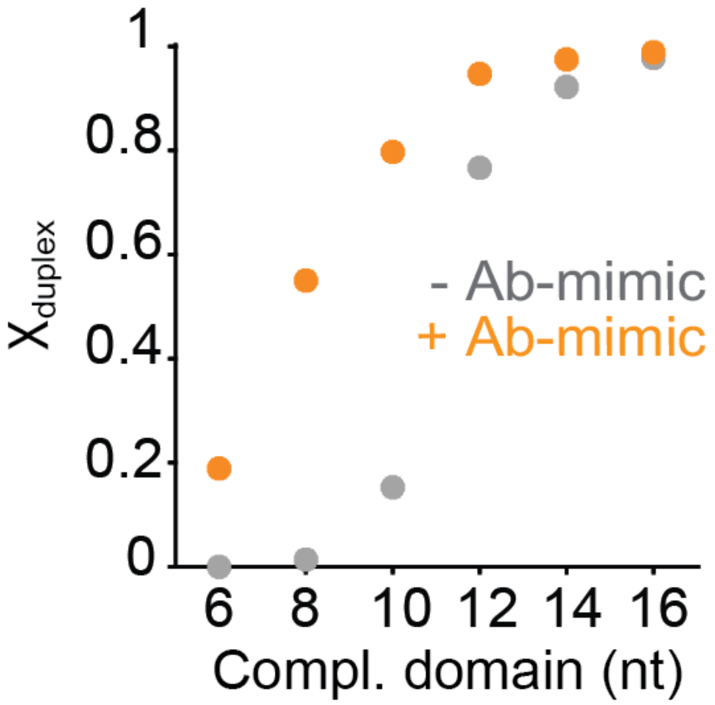

Supplementary Figure 6. ***Molar fraction of duplex formed in the presence and in the absence of Ab-mimic strand***. *Values were calculated for an equimolar concentration of templating pairs and Ab-mimic strand of 10^{-7} M, for all the different complementary lengths. Data from Supplementary Table 4.*

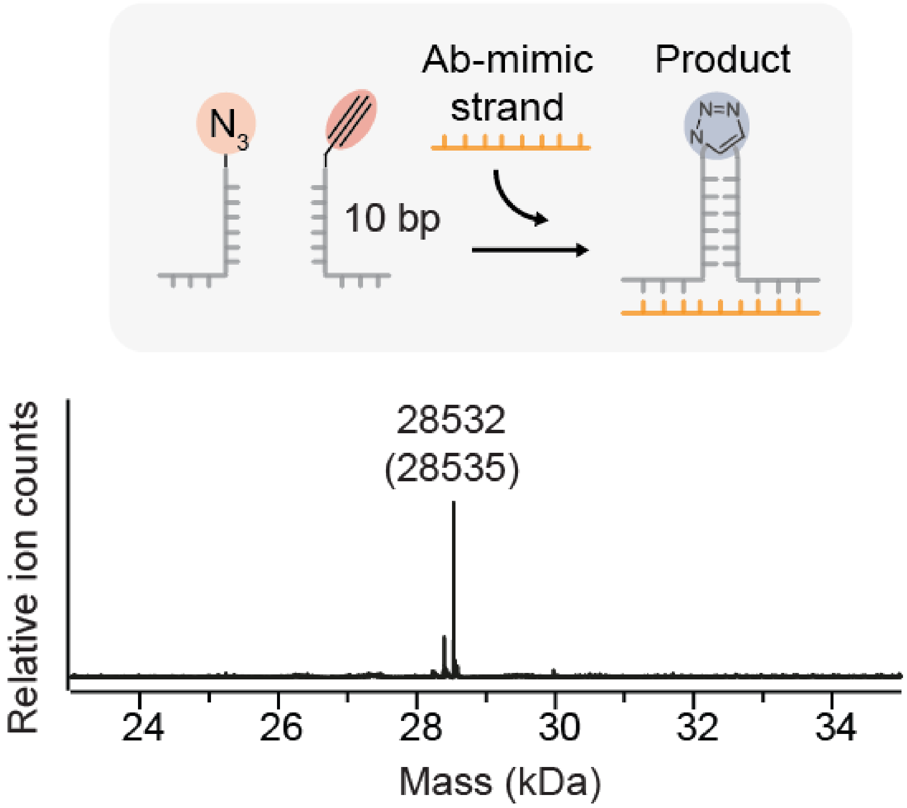

Supplementary Figure 7. *Deconvoluted ESI (−) mass spectrum of product from CuAAC reaction of 10-nt templating strands in presence of Ab-mimic strand. Observed and expected (in brackets) m/z values are indicated.*

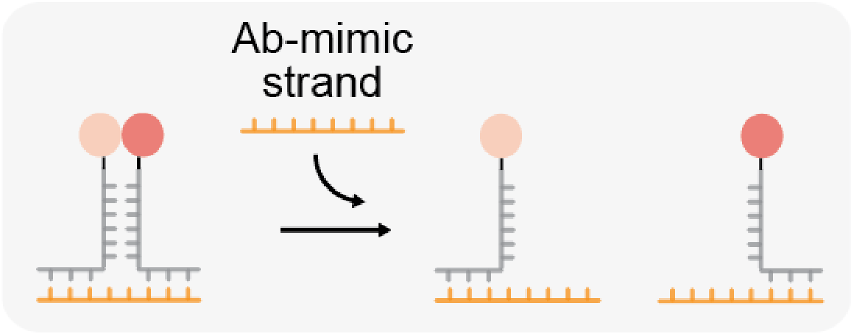

Supplementary Figure 8. *Excess concentration of Ab-mimic strand (i.e. 1 μM) produces a lower product yield likely due to the de-hybridization of the template duplex induced by the high concentration of Ab-mimic strand employed.*

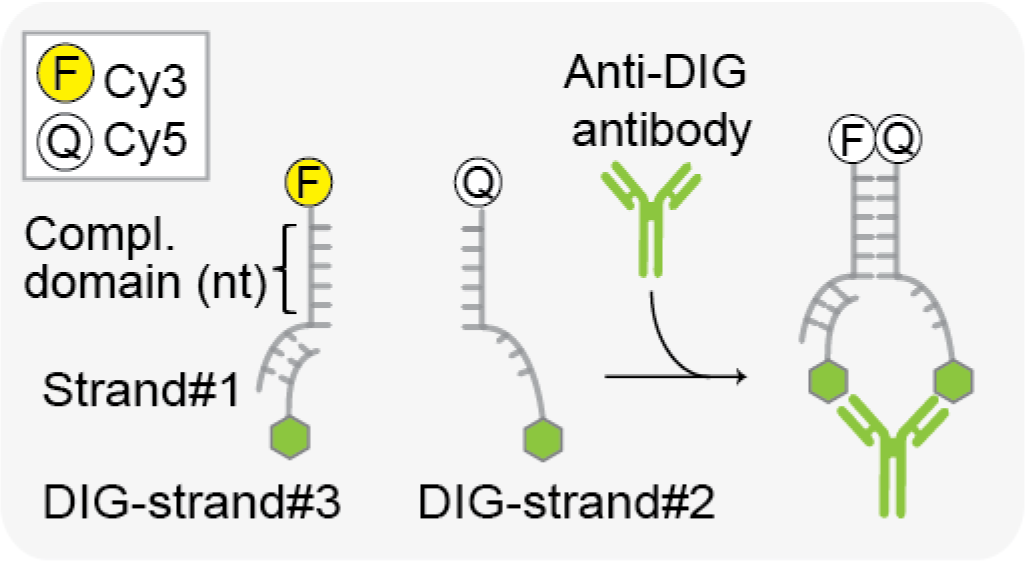

Supplementary Figure 9. ***Scheme of design used to study the effect of anti-DIG antibody on the duplex formation.*** *Optically and antigen-modified oligonucleotides were employed. Templating pairs with complementary domains of different length were tested.*

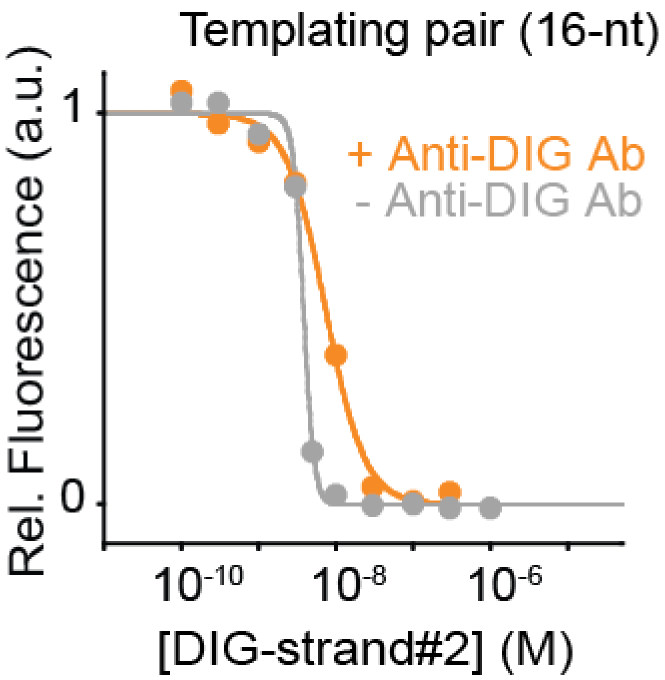

Supplementary Figure 10. ***Binding curves of the templating strands with a 16-nt complementary portion in the presence and absence of anti-DIG antibody.*** *Fluorescent experiments were performed at 37ºC in 25 mM HEPES buffer pH 7.2, 0.1 M NaCl at a fixed concentration of Strand#1 and DIG-strand#3 (10 nM) and increasing concentrations of DIG-strand#2 in the absence or presence (30 nM) of anti-DIG antibody.*

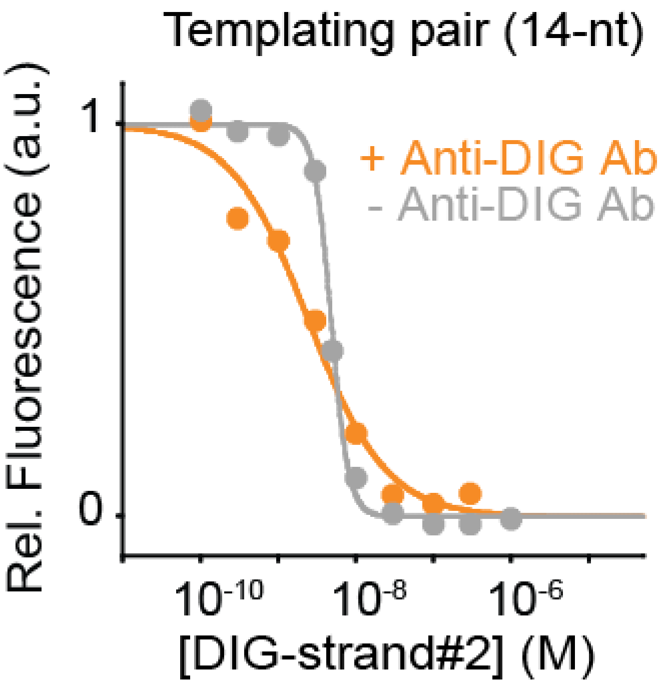

Supplementary Figure 11. ***Binding curves of the templating strands with a 14-nt complementary portion in the presence and absence of anti-DIG antibody.*** *Fluorescent experiments were performed at 37ºC in 25 mM HEPES buffer pH 7.2, 0.1 M NaCl at a fixed concentration of Strand#1 and DIG-strand#3 (10 nM) and increasing concentrations of DIG-strand#2 in the absence or presence (30 nM) of anti-DIG antibody.*

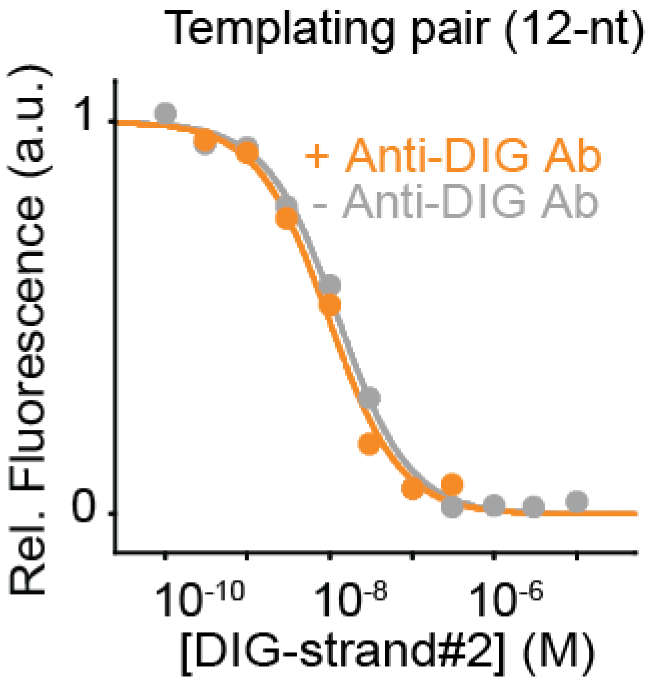

Supplementary Figure 12. ***Binding curves of the templating strands with a 12-nt complementary portion in the presence and absence of anti-DIG antibody.*** *Fluorescent experiments were performed at 37ºC in 25 mM HEPES buffer pH 7.2, 0.1 M NaCl at a fixed concentration of Strand#1 and DIG-strand#3 (10 nM) and increasing concentrations of DIG-strand#2 in the absence or presence (30 nM) of anti-DIG antibody.*

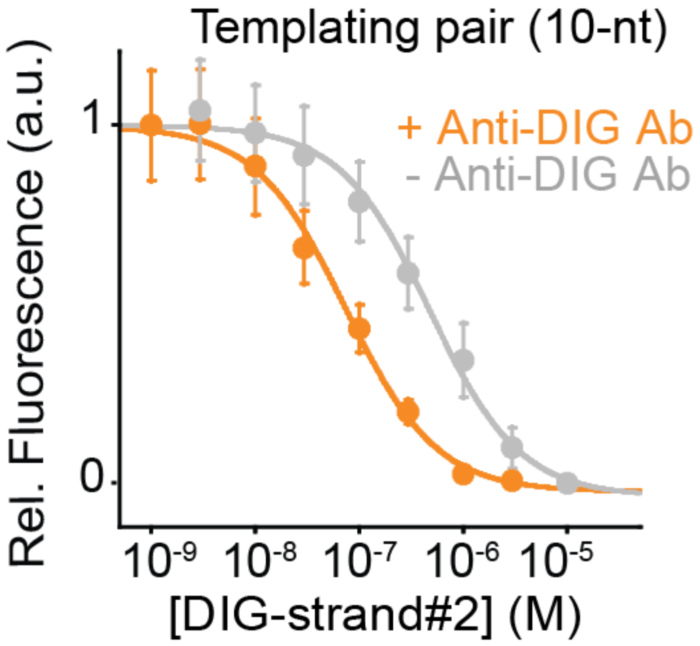

Supplementary Figure 13. ***Binding curves of the templating strands with a 10-nt complementary portion in the presence and absence of anti-DIG antibody.*** *Fluorescent experiments were performed at 37ºC in 25 mM HEPES buffer pH 7.2, 0.1 M NaCl at a fixed concentration of Strand#1 and DIG-strand#3 (10 nM) and increasing concentrations of DIG-strand#2 in the absence or presence (30 nM) of anti-DIG antibody. The experimental values represent averages of three separate measurements and the error bars reflect the standard deviations.*

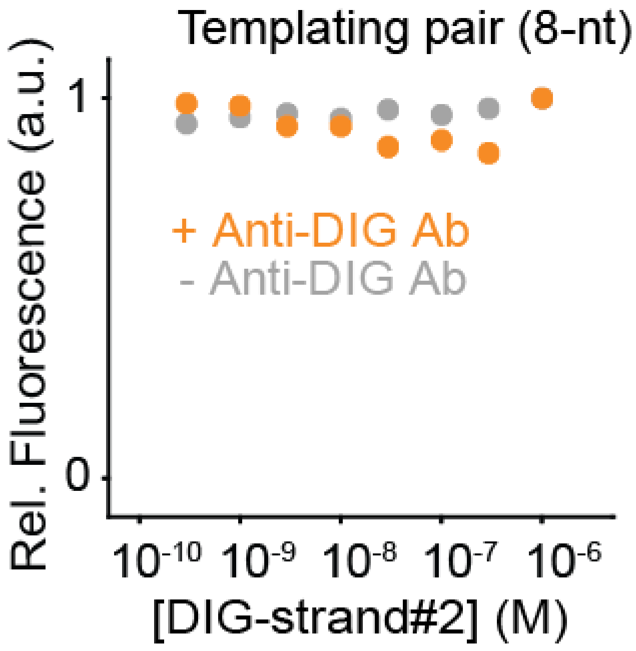

Supplementary Figure 14. *Binding curves of the templating strands with a 8-nt complementary portion in the presence and absence of anti-DIG antibody.* *Fluorescent experiments were performed at 37ºC in 25 mM HEPES buffer pH 7.2, 0.1 M NaCl at a fixed concentration of Strand#1 and DIG-strand#3 (10 nM) and increasing concentrations of DIG-strand#2 in the absence or presence (30 nM) of anti-DIG antibody.*

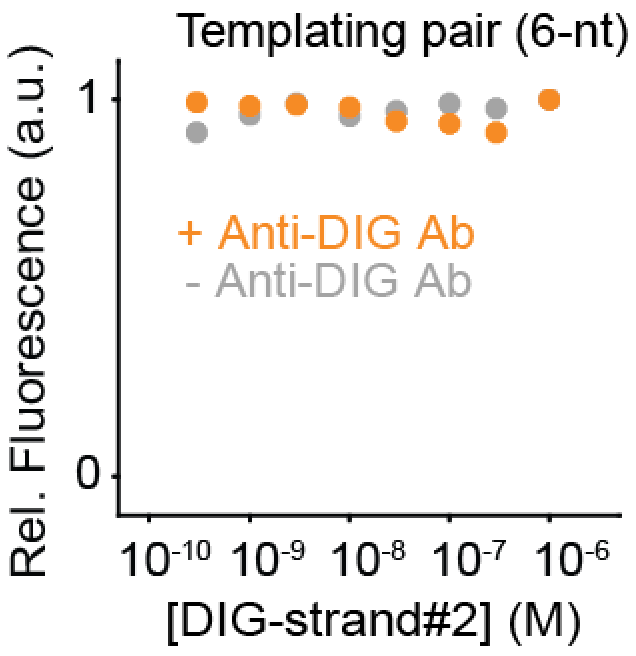

Supplementary Figure 15. ***Binding curves of the templating strands with a 6-nt complementary portion in the presence and absence of anti-DIG antibody.*** *Fluorescent experiments were performed at 37ºC in 25 mM HEPES buffer pH 7.2, 0.1 M NaCl at a fixed concentration of Strand#1 and DIG-strand#3 (10 nM) and increasing concentrations of DIG-strand#2 in the absence or presence (30 nM) of anti-DIG antibody.*

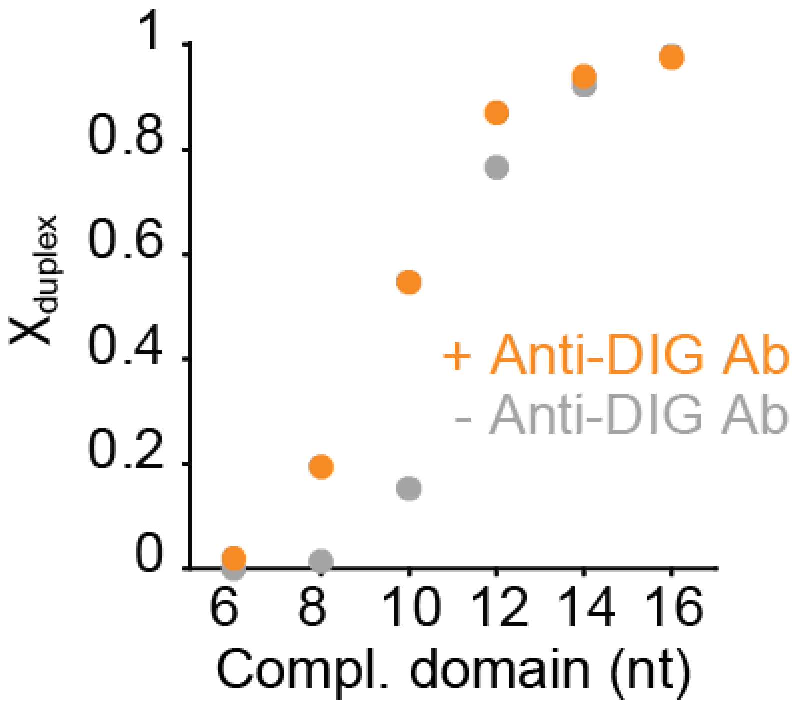

Supplementary Figure 16. ***Molar fraction of duplex formed in the presence and in the absence of anti-DIG antibody****. Values were calculated for an equimolar concentration of templating pairs and antibody of 10^{-7} M, for all the different complementary lengths. Data from Supplementary Table 6.*

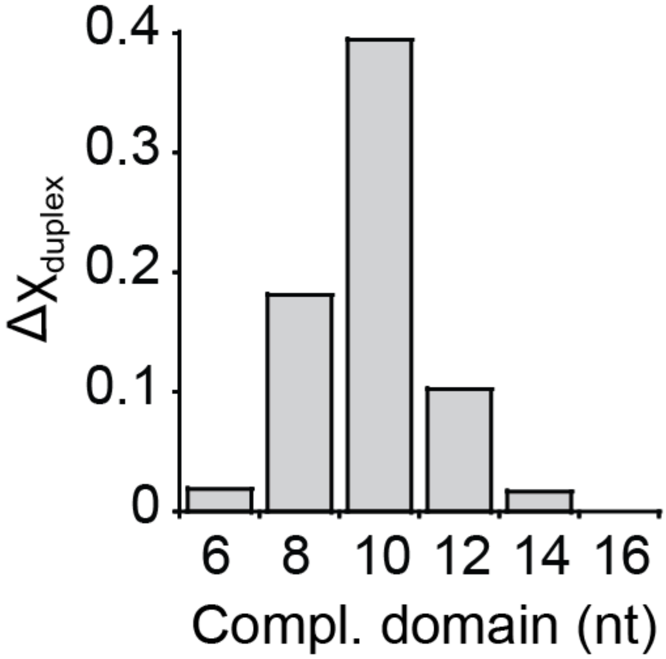

Supplementary Figure 17. *Difference in duplex molar fraction for different lengths of the complementary domain in the presence and absence of anti-DIG antibody calculated for an equimolar concentration of templating pairs and Ab-mimic strand of 100 nM. Data from Supplementary Table 6.*

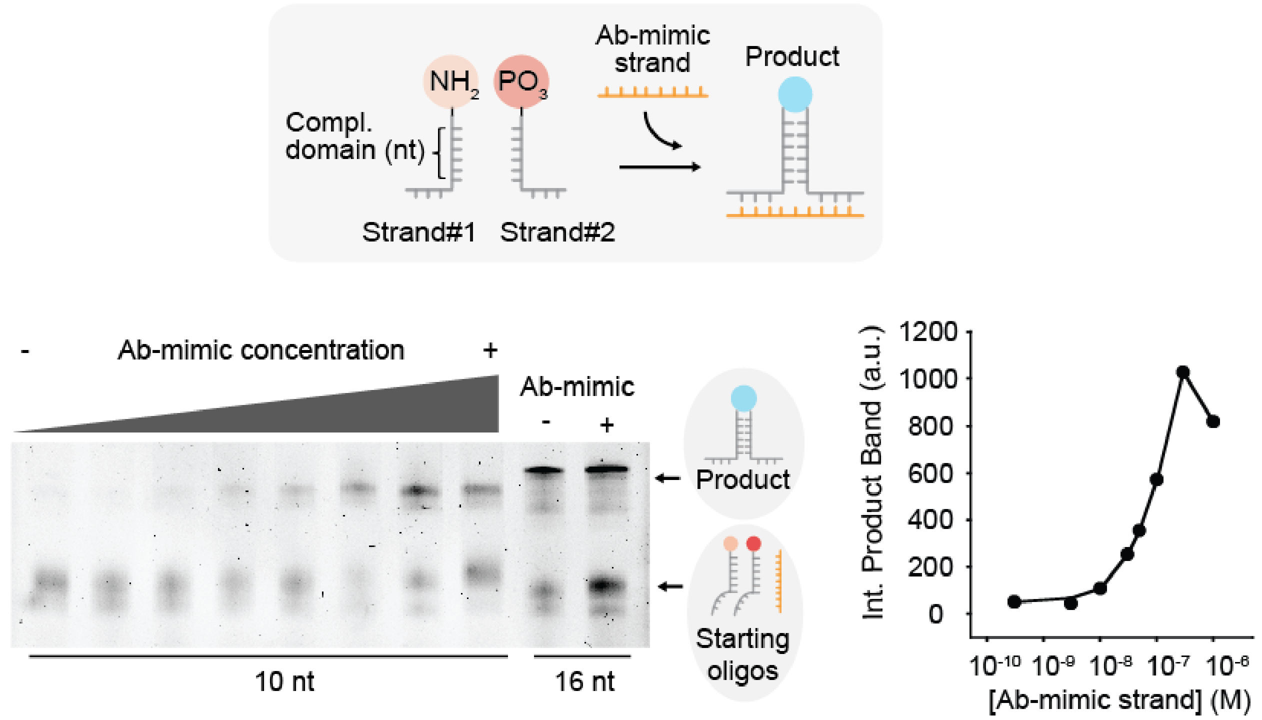

Supplementary Figure 18. ***Ab-mimic strand-templated phosphoramidate ligation.*** *The 5'-amino modified (NH_2-strand#1), 3'-phosphate modified (Phos-strand#2) and unmodified (Ab-mimic strand) oligos were combined at a concentration of 100 nM in a buffer containing 25 mM HEPES pH 7.2; 0.1 M NaCl. Reaction was initiated by the addition of 1 ul of 0.25 M EDC.HCl (final 25 mM) and 1 ul of 1 M 1-(2-hydroxyethyl) imidazole (final 100 mM) and left 2 hours at 37˚C without shaking. The total volume of the reaction was 10 ul.*

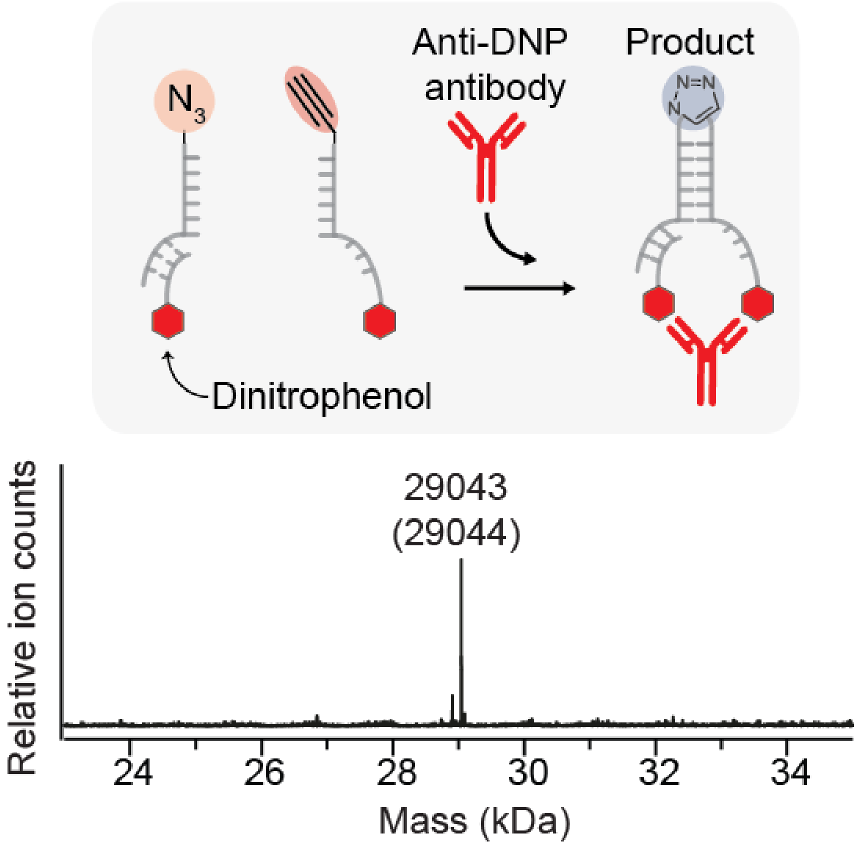

Supplementary Figure 19. *Deconvoluted ESI (−) mass spectrum of product from CuAAC reaction of 10-nt templating strands in presence of anti-DNP antibody. Observed and expected (in brackets) m/z values are indicated.*

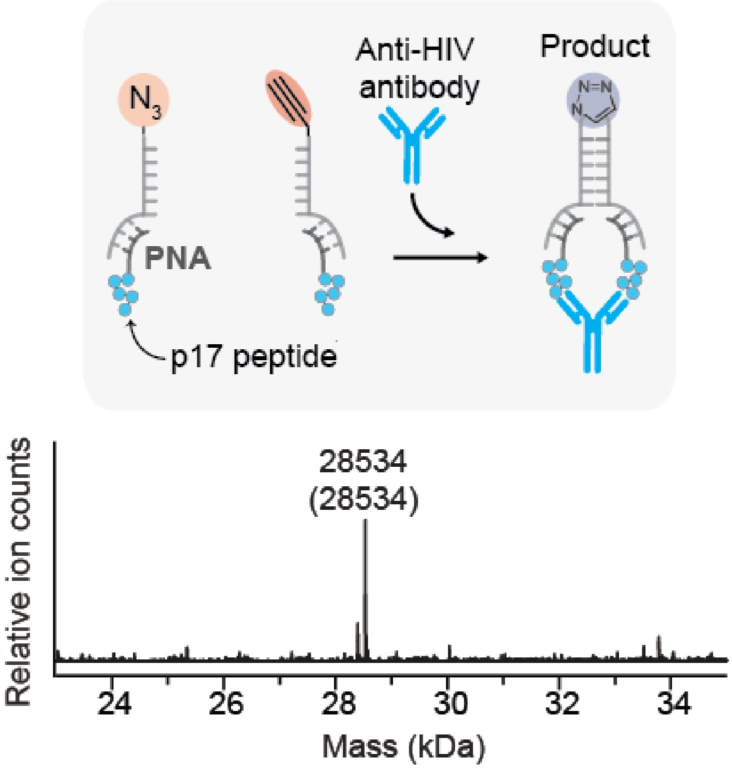

Supplementary Figure 20. *Deconvoluted ESI (−) mass spectrum of product from CuAAC reaction of 10-nt templating strands in presence of anti-HIV antibody. Observed and expected (in brackets) m/z values are indicated.*

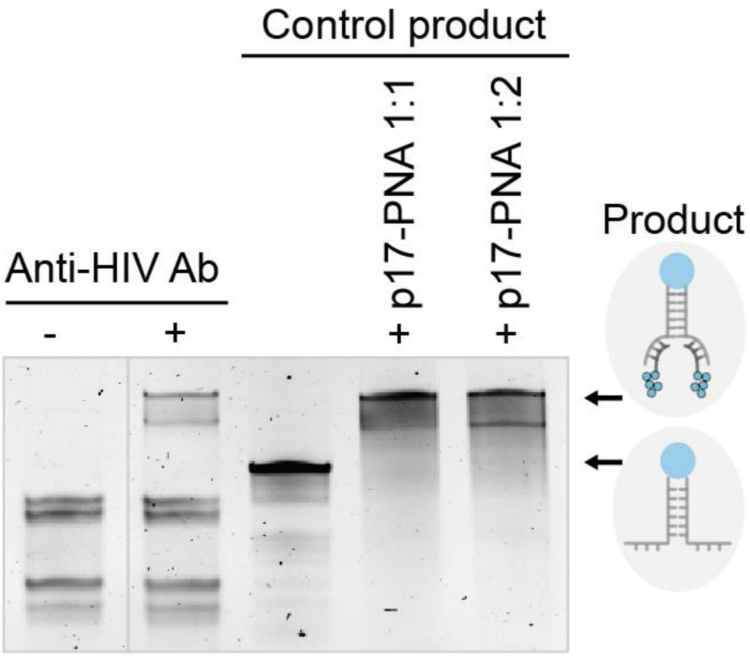

Supplementary Figure 21. C*hange in migration of product band due to its binding to the PNA sequence modified with peptide p17.* *The product band from the reaction experiences a change in migration (lane 2) compared to the product control (lane 3) due to the binding to the PNA sequence. The affinity of DNA-PNA duplexes is very high (higher than that of DNA-DNA duplexes)[3] and they could not be denatured even under the denaturing conditions used for PAGE. Templated reactions were performed at 37ºC for 2 h at a 100 nM concentration of templating strands and 300 nM of anti-HIV antibody in presence of Cu(l) catalyst in 25 mM HEPES buffer pH 7.2, 0.1 M NaCl.*

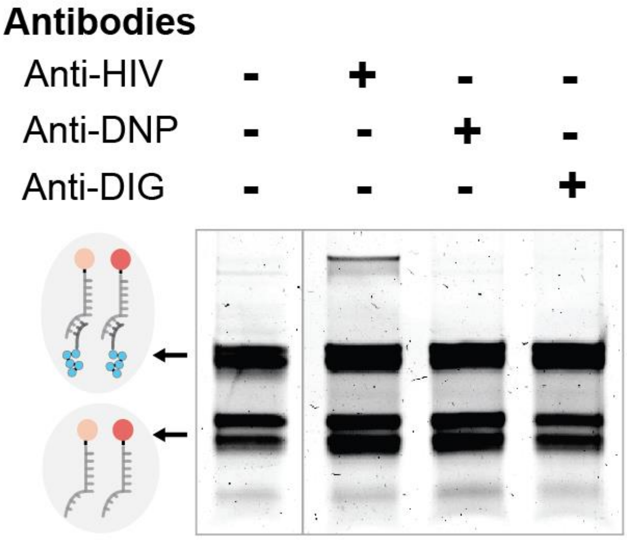

Supplementary Figure 22. *Specificity test of the reaction triggered by anti-HIV antibody.* *The reaction was carried out in the presence of the non-specific antibodies anti-DIG and anti-DNP. Templated reactions were performed at 37ºC for 2 h at a 100 nM concentration of templating strands and 300 nM of the corresponding antibody in presence of Cu(I) catalyst in 25 mM HEPES buffer pH 7.2, 0.1 M NaCl.*

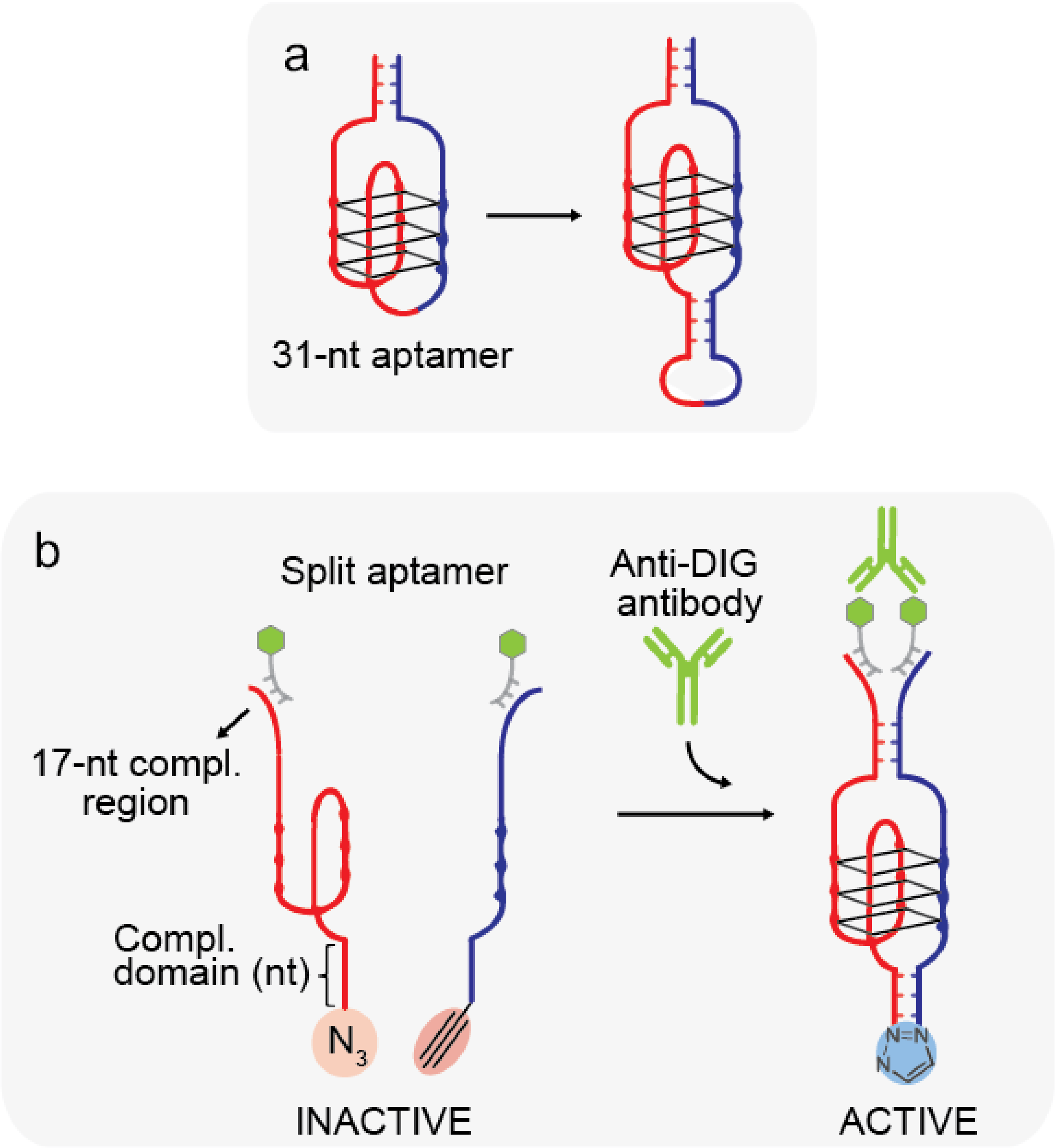

Supplementary Figure 23. ***Design of the inactive and active forms of the thrombin-inhibiting aptamer. a)*** *Original 31-nt thrombin-inhibiting aptamer (left) and the resulting structure after inserting a stem-loop within the G-quadruplex structure (right) by Ikebukuro and co-workers*[1]. ***b)*** *Split aptamer (inactive form) modified with CuAAC reactive groups leads to the active form of the aptamer upon binding to anti-DIG antibody and subsequent reaction.*

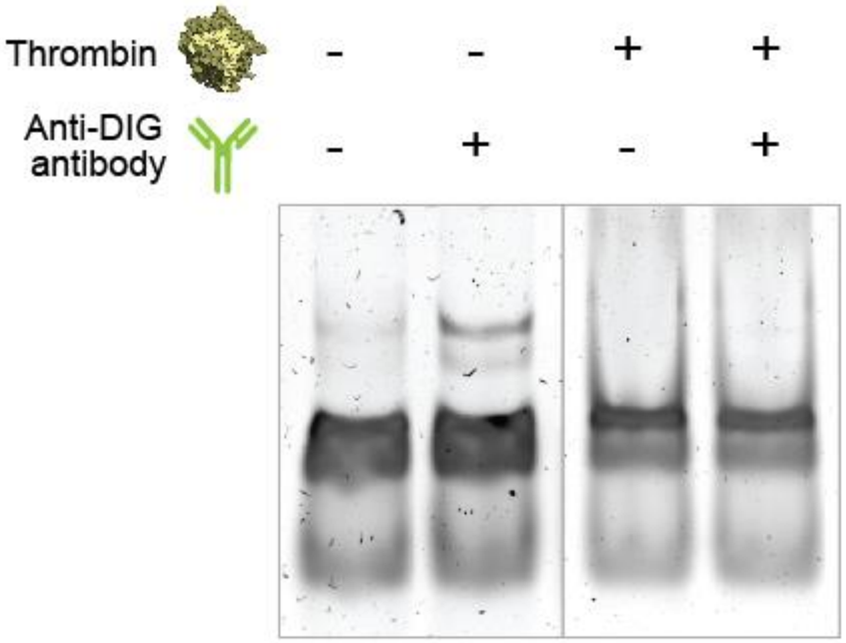

Supplementary Figure 24. Native PAGE (8%) assay to study aptamer-thrombin binding. *Templated reactions were performed at 37ºC for 2 h at a 100 nM concentration of templating strands and 300 nM of anti-DIG antibody in presence of Cu(I) catalyst in 25 mM HEPES buffer pH 7.2, 0.1 M NaCl. Reactions were followed by heat treatment at 95ºC for 3 min to denature the antibody. Thrombin (1 µM) was added to the solution (when it applies), incubated for 15 min to allow for binding and samples were loaded into the gel.*

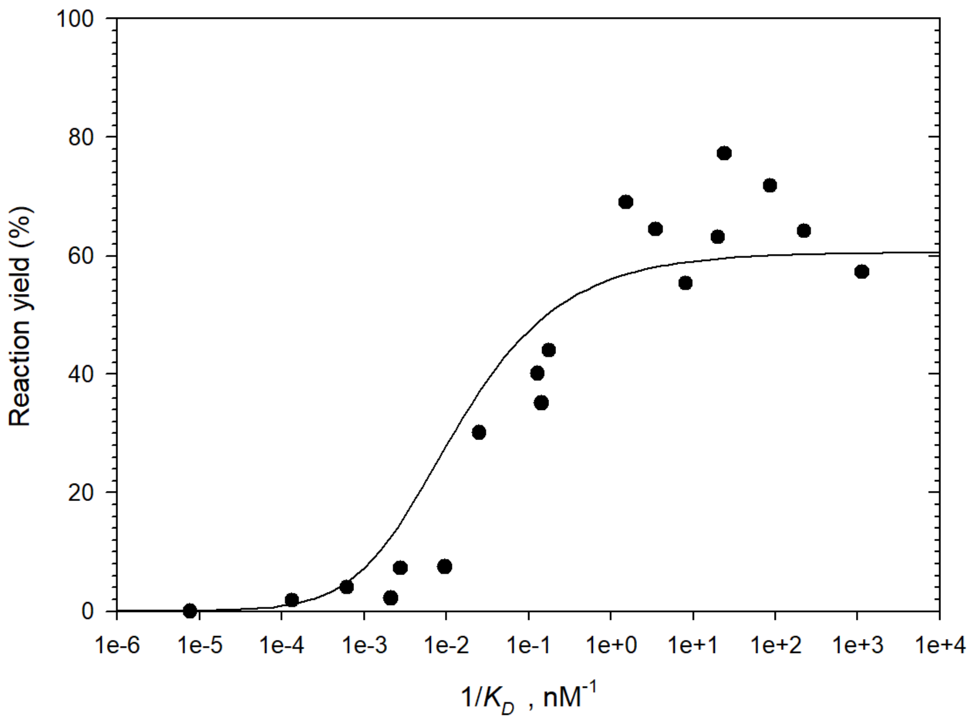

Supplementary Figure 25. *Plot of the reaction yield as a function of 1/K_D, calculated by equation (3) in the main text, under the conditions c_0 = 100 nM, k = 0.465 h^{-1}, and t = 2 h. The solid curve is obtained by fitting* equation (3) *to the experimental yield values from Supplementary Tables 7-9.*

Supplementary Table 1. Predicted Gibbs free energies for strand hybridization

Length of complementary domains (nt)	$-\Delta G_{pred}$ (kcal/mol)
16	28.54
14	25.37
12	22.45
10	17.26
8	13.85
6	10.33

Supplementary Table 2. Values of the dissociation constants for DNA duplexes of different complementary lengths in the absence of any added template.

Length of complementary domains (nt)	$K_{D\_no\_template}$ (M)
16	5.0×10^{-11} [a]
14	6.5×10^{-10} [a]
12	7×10^{-9} [b]
10	4.7×10^{-7} [b]
8	7.5×10^{-6} [a]
6	1.3×10^{-4} [a]

[a] Estimated by equation (S4). [b] Experimental value

Supplementary Table 3. Values of the apparent dissociation constants for DNA templated duplexes of different complementary lengths in the presence of [*Ab_mimic*] = 10^{-7} M.

Length of complementary domains (nt)	$K_{D\_Ab\_mimic}$ (M)
16	1.1×10^{-13}
14	1.4×10^{-12}
12	1.5×10^{-11}
10	1.0×10^{-9}
8	1.7×10^{-8}
6	2.9×10^{-7}

Supplementary Table 4. Duplex molar fractions in the absence and presence of the Ab-mimic strand at the equimolar concentration of templating pairs, and Ab-mimic strand, of 10^{-7} M.

Length of complementary domains (nt)	$x_{no\_template}$	x_{Ab_mimic}	Δx
16	0.978	0.990	0.012
14	0.923	0.976	0.053
12	0.768	0.947	0.179
10	0.153	0.798	0.645
8	1.30×10^{-2}	0.550	0.538
6	7.68×10^{-4}	0.188	0.187

Supplementary Table 5. Values of the apparent dissociation constants for DNA templated duplexes of different complementary lengths in the presence of [Anti-DIG antibody] = 10^{-7} M.

Length of complementary domains (nt)	$K_{D\_Anti\_DIG\_Ab}$ (M)
16	1.8×10^{-12}
14	2.3×10^{-11}
12	2.5×10^{-10}
10	1.7×10^{-8}
8	2.7×10^{-7}
6	4.7×10^{-6}

Supplementary Table 6. Duplex molar fractions in the absence and presence of the anti-DIG antibody at the equimolar concentration of templating pairs, and anti-DIG antibody, of 10^{-7} M.

Length of complementary domains (nt)	$x_{no\_template}$	$x_{Anti\_DIG\_Ab}$	Δx
16	0.978	0.974	≈ 0
14	0.923	0.940	0.017
12	0.768	0.870	0.102
10	0.153	0.548	0.395
8	1.30×10^{-2}	0.194	0.181
6	7.68×10^{-4}	0.020	0.019

Supplementary Table 7. Experimental reaction yields of templating pairs in the absence of any template, and corresponding $K_{D\_no\_template}$ values from Supplementary Table 2.

Length of complementary domains (nt)	*Reaction yield (%)*	$K_{D\_no\_template}$ (M)
16	63.1	5.0×10^{-11}
14	69	6.5×10^{-10}
12	35.1	7×10^{-9}
10	2.2	4.7×10^{-7}
8	1.8	7.5×10^{-6}
6	0	1.3×10^{-4}

Supplementary Table 8. Experimental reaction yields of templating pairs in the presence of Ab-mimic strand at the calculated average free concentration, and corresponding $K_{D\_Ab\_mimic}$ values calculated by equation (S6).

Length of complementary domains (nt)	*Reaction yield (%)*	[*Ab_mimic*]$_{avg}$ (nM)	$K_{D\_Ab\_mimic}$ (M)
16	64.2	2.4 ± 1.4	4.5×10^{-12}
14	77.3	3.4 ± 1.0	4.2×10^{-11}
12	64.5	5.4 ± 0.1	2.9×10^{-10}
10	44.0	18 ± 2	5.7×10^{-9}
8	30.1	41 ± 4	4.0×10^{-8}
6	7.3	79 ± 3	3.6×10^{-7}

Supplementary Table 9. Experimental reaction yields of templating pairs in the presence of anti-DIG antibody at the calculated average free concentration, and corresponding $K_{D\_Anti\_DIG\_Ab}$ values calculated by equation (S14).

Length of complementary domains (nt)	*Reaction yield (%)*	$[Anti\_DIG\_Ab]_{avg}$ (nM)	$K_{D\_Anti\_DIG\_Ab}$ (M)
16	57.3	2.0×10^{2}	8.9×10^{-13}
14	71.8	2.0×10^{2}	1.2×10^{-11}
12	55.3	2.0×10^{2}	1.2×10^{-10}
10	40.1	2.2×10^{2}	7.8×10^{-9}
8	7.5	2.6×10^{2}	1.0×10^{-7}
6	4.0	2.9×10^{2}	1.6×10^{-6}

Supplementary References

1. Yoshida, W., Sode, K. & Ikebukuro, K. Homogeneous DNA sensing using enzyme-inhibiting DNA aptamers. *Biochem. Biophys. Res. Commun.* **348**, 245–252 (2006).
2. Owczarzy, R. *et al.* IDT SciTools: a suite for analysis and design of nucleic acid oligomers. *Nucleic Acids Res.* **36**, 163–169 (2008).
3. Gupta, A., Mishra, A. & Puri, N. Peptide nucleic acids: Advanced tools for biomedical applications. *J. Biotechnol.* **259**, 148–159 (2017).
